# UV-Vis Spectroelectrochemistry of Oleuropein, Tyrosol, and *p*-Coumaric Acid Individually and in an Equimolar Combination. Differences in LC-ESI-MS^2^ Profiles of Oxidation Products and Their Neuroprotective Properties

**DOI:** 10.3390/biom9120802

**Published:** 2019-11-28

**Authors:** Morgane Lambert de Malezieu, Solenn Ferron, Aurélie Sauvager, Patricia Courtel, Charles Ramassamy, Sophie Tomasi, Marie-Laurence Abasq

**Affiliations:** 1 Univ. Rennes, CNRS, ISCR–UMR 6226, 35043 Rennes, France; lam2maz@yahoo.fr (M.L.d.M.); solenn.ferron@univ-rennes1.fr (S.F.); aurelie.sauvager@univ-rennes1.fr (A.S.); 2 INRS-Centre Armand Frappier, Laval, QC H7V 1B7, Canada; patricia.courtel@univ-rennes1.fr (P.C.); Charles.Ramassamy@iaf.inrs.ca (C.R.); 3 INAF, Québec, QC G1V 0A6, Canada

**Keywords:** olive oil phenolic compounds, UV-Vis spectroelectrochemistry, LC/MS^2^, oxidation, electron transfer, neuroprotection

## Abstract

Major phenolic compounds from olive oil (ArOH-EVOO), oleuropein (Ole), tyrosol (Tyr), and *p*-coumaric acid (*p*-Cou), are known for their antioxidant and neuroprotective properties. We previously demonstrated that their combination could potentiate their antioxidant activity in vitro and in cellulo. To further our knowledge of their electron-transfer properties, Ole, Tyr, and *p*-Cou underwent a spectroelectrochemical study, performed either individually or in equimolar mixtures. Two mixtures (Mix and Mix-seq) were prepared in order to determine whether distinct molecules could arise from their simultaneous or sequential oxidation. The comparison of Liquid Chromatography–Electrospray Ionization–Tandem Mass Spectrometry (LC-ESI-MS^2^) profiles highlighted the presence of specific oxidized products found in the mixes. We hypothesized that they derived from the dimerization between Tyr and Ole or *p*-Cou, which have reacted either in their native or oxidized forms. Moreover, Ole regenerates when the Mix undergoes oxidation. Our study also showed significant neuroprotection by oxidized Ole and oxidized Mix against H_2_O_2_ toxicity on SK-N-SH cells, after 24 h of treatment with very low concentrations (1 and 5 nM). This suggests the putative relevant role of oxidized Ole products to protect or delay neuronal death.

## 1. Introduction

**Figure 1:**
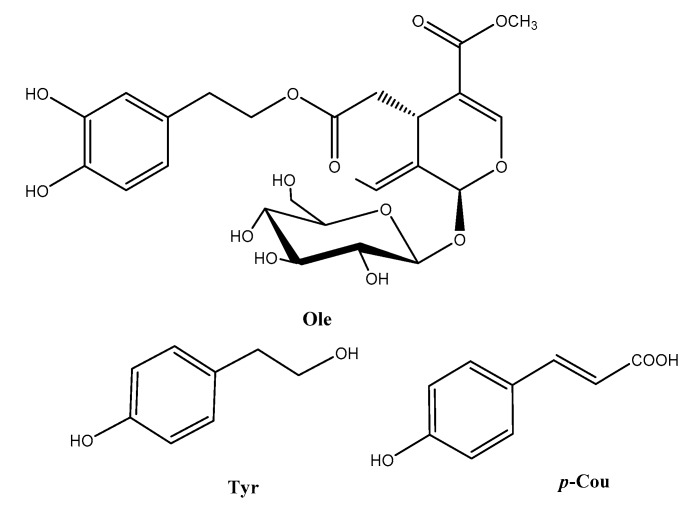
Structure of the studied ArOH-EVOO.

Phenolic compounds from olive oil (ArOH-EVOO) are studied for their antioxidant and neuroprotective properties. Indeed, the *ortho*-diphenol oleuropein (Ole) and its derivative hydroxytyrosol (OH-Tyr) have shown some interesting reactivities in non-cellular in vitro assays, such as DPPH (2,2-diphenyl-1-picrylhydrazyl), superoxide, peroxyl radicals inhibition, and reducing capacity methods [1,2,3] (Figure 1). The chemistry behind these assays involves H-atom transfer (HAT), radical-chain breaking, and electron transfer (ET) mechanisms, which have been detailed by Apak [4]. The monophenols, tyrosol (Tyr), and *p*-coumaric acid (*p*-Cou) are weaker H-atom and electron donors, even though *p*-Cou could reveal superoxide scavenging capacity almost as high as Ole and OH-Tyr ascribed to its higher conjugative effects [5,6,7,8,9]. In these non-cellular assays, the reactivity of the ArOH-EVOO engages among others the formation of oxidized species, which have been partially analyzed so far by NMR or HPLC-MS. Scarce molecules have been fully identified as quinones, dimers, trimers, aldehydes, or acids [2,10,11,12,13,14,15,16]. Moreover, ArOH-EVOO can act on cell signaling pathways and have also shown some antioxidant effects in cellular models by activation or inactivation of redox factors when, for example, neuronal cells are exposed to chemical stressors [17,18,19,20]. The biochemistry behind the regulation is not fully understood and could involve oxidized metabolites. An interesting highlight is that some molecule combinations enhance the antioxidant efficacy in both non-cellular and cellular in vitro assays by synergic effects [1,21,22,23]. Indeed, our previous work has shown for instance that a low concentration equimolar mix of Ole, Tyr, and *p*-Cou (0.1 and 1 µM) reduces intracellular ROS and protein carbonyl levels, and prevents the activation of Nrf2 and NF-κB in neuronal SK-N-SH cells challenged with H_2_O_2_ or Paraquat [9]. This is much lower than the concentration of any one individual molecule required to observe similar effects. In this previous study, also evaluating superoxide or hydrogen peroxide inhibition, a significant difference in reactivity of Mixes was observed. Indeed the presence of the catechol Ole in combination with the two monophenols (Tyr, *p*-Cou) displayed higher reactivity than when the catechol hydroxytyrosol was present [9]. The enhancement of the activity was attributed to possible coupled redox and intermolecular reactions generating stronger H-atom or electron donor compounds when Ole is present. In this present study, we propose an original approach to explore this assumption by electrochemically oxidizing the three molecules, either simultaneously or sequentially, and employing LC-ESI-MS^2^ to determine whether new molecules emerge from the combination.

So far, glucuronidated, sulfated or methylated ArOH-EVOO metabolites are well known and studied in both in vivo and ex vivo studies [24,25,26], but do not exclude the presence of other types of metabolites issued from a redox pathway as it was demonstrated by Fasi et al. [27]. It has also been suggested that oxidation products of Tyr, presenting few in vitro antioxidant capacities under its native form, could be responsible for its cellular effect in neuronal cells, notably to explain the regulation of Nf-κB [17]. A recent review emphasized, thus, the roles of all metabolites included the oxidized ones in the regulation of redox signaling pathways [28].

In this study, the objective was twofold. A spectroelectrochemical technique was used to access stably oxidized molecules of ArOH-EVOO (Ole, Tyr, and *p*-Cou) (Figure 1), studied singly or in combination. This helped update the liquid chromatography-tandem mass spectrometry (LC-ESI-MS^2^) database, which will be helpful to understand the possible role of these molecules in cell signaling pathways. Moreover, we focused on the differences in LC-ESI-MS^2^ profiles of oxidation products between all samples studied. Moreover, the neuroprotective effects of some oxidized samples were evaluated to thwart H_2_O_2_ induced neuronal death.

## 2. Materials and Methods

### 2.1. Chemicals and Reagents

Commercially available chemicals were used without any further purification. Tyrosol, *p*-coumaric acid, and oleuropein were obtained as analytical standards (≥98%, HPLC) from Sigma-Aldrich (Oakville, ON, Canada). Phosphate-buffered saline (PBS) pH 7.4 0.1 M was purchased from Lonza (Basel, Switzerland). MilliQ water was used for all the experiments. Oleuropein, tyrosol, and *p*-coumaric acid were dissolved in DMSO then diluted in PBS to obtain adequate concentrations for square wave voltammetry (SWV) and electrolysis procedures (5% DMSO final concentrations). A blend of Ole, Tyr, and *p*-Cou, called Mix, was prepared in equimolar proportion at 2 mM or 10 µM total concentration in non-cellular and cellular assays, respectively. A sequential mixture, coded Mix-seq, was also prepared and electrolyzed following the procedure described below (see 2.2.6).

### 2.2. Electrochemical Procedures

#### 2.2.1. Square Wave Voltammetry (SWV) of Phenolic Compounds

SWV experiments were carried out on a dual potentio-galvanostat PGSTAT100 (Autolab instrument, Eco Chemie B.V., Utrecht, The Netherlands). Measurements were performed with a three-electrode thermostated cell. A glassy carbon disk working electrode (Ø2 mm), a platinum wire counter electrode and a reference electrode, Ag/AgCl/KCl 3 M, were used. Before each measurement, the glassy carbon disk working electrode was polished on a waterproof Silicon Carbide Paper 4000 (Struers, Ballerup, Denmark) using a 0.3 µm alumina suspension, washed with distilled water and dried. For the second SWV scan, the electrode surface was not polished in order to investigate if new peaks would emerge, which would mean the formation of new compounds in the diffusion layer following the first scan. The temperature was maintained at 20 ± 0.02 °C with a Julabo heating circulator MP-5 (Julabo, Seelbach, Germany). Before each measurement, a 25 µM ArOH solution was freshly prepared in 0.1 M phosphate buffer pH 7.4 and nitrogen saturated during 10 min. The SWV was recorded at a scan rate 0.05 V s^−1^. E*_pa_* and E*_pc_* are relative to anodic and cathodic potential, respectively. i, i_t_, i_f_, i_b_ are relative to current intensity, total current, forward current, backward current, respectively.

#### 2.2.2. Spectroelectrochemical Measurements

Spectroelectrochemical studies were conducted using a combined potentiostat/galvanostat (AUTOLAB PGSTAT 204, Metrohm Autolab, Utrecht, The Nederlands) and spectrometer (Avantes, AvaSpec-ULS20 48, Apeldoorn, The Netherlands) equipped with a UV-Visible deuterium/halogen light source (200–2500 nm). This system was controlled by NOVA 1.10 software (Metrohm Autolab). The three-electrode cell was used comprising a platinum grid working electrode (7 × 6 mm, wire diameter 0.3 mm), a platinum wire counter electrode and an Ag/AgCl/KCl 3M reference electrode placed in a spectrophotometric quartz cuvette (SEC-C thin layer quartz cristal spectroelectrochemical cell, 1 mm optical path length (ALS CO., Ltd. Tokyo, Japan). All potentials reported herein are *vs*. Ag/AgCl reference electrode. Before each measurement, the platinum grid working electrode was washed with H_2_SO_4_ 0.2 M and sonicated for 5 min. Then, cleaning was done by recording 200 cyclic voltammograms in H_2_SO_4_ 0.2 M between −0.5 V and +1.5 V (scan rate 0.5 V s^−1^). In a second step, the platinum grid was cleaned, recording 200 cyclic voltammograms in phosphate buffer pH 7.4 between −1.2 and +1.5 V (scan rate 0.5 V s^−1^). Before all measurements, a 2 mM ArOH solution was freshly prepared in phosphate buffer pH 7.4 0.1 M and nitrogen saturated during 15 min at room temperature. For Mix and Mix-seq, the total phenolic concentration in an equimolar ratio of each compound was 2 mM. For each sample, an aliquot was immediately argon saturated and stored at −20 °C (reference sample), and an aliquot of 600 µL was collected for the amperometry experiment. After electrolysis, the whole electrolyzed solution was collected, argon saturated in a sealed vial and stored at −20 °C.

#### 2.2.3. Linear Sweep Voltammetry (LSV)

The LSV (i = f (E)) were firstly recorded at a scan rate of 0.005 V s^−1^ in order to determine the anodic potential E_pa_ of the phenolic and Mixes samples. They were respectively measured at +0.32 V for Ole, +0.54 V for *p*-Cou, +0.59 V for Tyr, and +0.27 V, and +0.65 V for Mix. The UV-Vis spectra were simultaneously recorded between 200 and 600 nm (see Figure 3).

#### 2.2.4. Electrolysis

Electrolysis were performed by a chronoamperometry technique i = f (t) at a fixed potential during 7200 s (see Section 2.2.5 and Section 2.2.6). The UV-Vis spectra were simultaneously recorded. During electrolysis, nitrogen bubbling was maintained, first to avoid any oxidation derived from oxygen and also to mix the solution in order to oxidize a maximum of phenolic molecules (the whole cell volume is 600 µL, whereas the optic way compartment holds approximatively 200 µL of the solution). After electrolysis, the solution was retrieved, saturated with argon in a sealed vial and stored at −20 °C.

#### 2.2.5. Electrolysis of Ole, Tyr, and *p*-Cou Individually

The applied electrolysis potential was fixed at +0.7 V for the Ole electrolysis, +1 V for the Tyr, and *p*-Cou electrolysis and +0.90 V for Mix.

#### 2.2.6. Simultaneous and Sequential Electrolysis of Ole, Tyr and *p*-Cou in Combination

The potential was fixed at +0.90 V for the Mix electrolysis. For Mix-seq procedure, a 1:1 (*v*/*v*) blend of 2 mM tyrosol and 2 mM *p*-coumaric acid was freshly prepared in PBS and electrolyzed at +0.90 V during 7200 s. An identical volume of 2 mM oleuropein in PBS was added in this electrolyzed sample to obtain a reference, which was stored at −20 °C under argon until LC-MS analysis. It was labelled as [(Tyr + *p*-Cou) Ox + Ole] or as Mix-seq. In a (Tyr, *p*-Cou) Ox sample, Ole was added, and the electrolysis was prolonged during 7200 sec at +0.7 V, then retrieved and stored. It was coded as Mix-seq Ox.

### 2.3. HPLC-DAD-ESI-MS^2^ Process

Reference and electrolyzed solutions, previously filtered on PTFE 0.45 µM filter (VWR, Fontenay-sous-Bois, France) were analyzed at 2 mM of reference solution on a system equipped with a SCM1000 degasser (ThermoScientific, San Jose, CA, USA), a 1100 series binary high-pressure pump (Agilent Technologies, Palo Alto, CA, USA), and a Surveyor autosampler thermostated at 4 °C (ThermoFinnigan, San Jose, CA, USA). Two detectors were connected in series: a UV-visible diode array detector (model UV6000 LP, ThermoFinnigan) and an Ion Trap Mass Spectrometer equipped with an electrospray ionization source (model LCQ Deca, ThermoFinnigan). The reversed-phase chromatography column used was a Purospher^®^ STAR RP-18 endcapped (3 µm) Hibar^®^ HR (Merck, Kenilworth, New Jersey, 2.1 × 150 mm) equipped with a precolumn Eclipse XDB-C8 (Agilent Technologies, 2.1 × 12.5 mm, 5 µm). The analysis procedure was adapted from Guyot et al. [29]. The oven was thermostated at 30 °C. Two microliters of sample were injected for analysis. The solvent system was a gradient of A (0.1% formic acid in ultrapure water) and B (0.1% formic acid in acetonitrile), which were filtered on a 0.45 μm GH Polypro membrane (VWR, Fontenay sous Bois, France), respectively, before being used. An elution gradient was applied as follows with a flow rate of 0.2 mL/min: initial, 3% B; 0–3 min, 7% B, linear; 3–21 min, 13% B, linear; 21–27 min, 13% B isocratic; 27–39 min, 38% B, linear; 39–47 min, 50% B, linear; 47–58 min, 90% B, linear; 58–61 min, 90% B; followed by washing and reconditioning the column. UV-visible detection covered the 240–600 nm wavelength range. Peaks corresponding to Tyr, *p*-Cou, and Ole were integrated at 275, 310, and 280 nm, respectively.

The whole effluent from the UV-Visible detector was injected in an ESI (Electrospray Ionization) source. The source parameters were negative ion mode, spray voltage (4.2 kV), capillary voltage (−41 V), sheath gas (66 arbitrary units), auxiliary gas (10 arbitrary units), and capillary temperature (250 °C). The nebulizing gas was nitrogen, and the damping gas was helium. MS spectra were acquired in full scan, negative ionization mode in the *m/z* 50−2000 range. For the MS^2^ experiment, collision energy of 35 eV was used. Data obtained were processed with the Xcalibur 1.2 software (Finnigan Corp., San Jose, CA, USA).

### 2.4. HPLC-DAD-ESI-MS^2^ Analysis

In order to identify the neoformed compounds, the UV-Vis chromatogram, and the MS spectrum of the electrolyzed solution were compared to their own reference. Particular attention was paid to wavelength maxima of the standard ArOH: 280 nm for Ole, Tyr, and OH-Tyr, and 320 for *p*-Cou. All experiments were carried out in triplicate. Native compounds were identified using their pure standards. Neoformed compounds following electrolysis were tentatively identified by combining their retention time (RT), mass spectra and fragmentation, and UV-Vis absorption maxima described in the cited literature.

### 2.5. Cell Culture and Cell Viability Assays

In order to determine if the oxidized phenolic compounds had neuroprotective capacities, 2 × 600 µL of Ole 10 µM or the Mix 10 µM were electrolyzed as previously described and then pooled. The electrolyzed solutions were then lyophilized before solubilization in PBS, in order to obtain a stock solution at 10 µM relative to the concentration of native compounds. Then, adequate dilution was done in the cell culture medium.

### 2.6. Cell Viability Assays

The human neuroblastoma SK-N-SH cells were maintained in MEM (Minimum Essential Media) supplemented with 10% (*v*/*v*) FBS (Fetal Bovine Serum), 100 µg/mL penicillin, 100µg/mL streptomycin, and 1% sodium pyruvate (1 mM) in a humidified incubator at 37 °C with 95% O_2_ and 5% CO_2_. Twenty four hours after seeding, cells were starved for 1 h before any treatment. Cells were grown to 80% confluence and then plated at a density of two E4 cells/well in 96-well plates and incubated for 24 h at 37 °C. Cell survival was assessed 24 h after the treatments, using the Tox-8 (Resazurin-based) kit following the manufacturer’s instructions. Measurements were done on six wells for three separated experiments, and the results were expressed as mean ± SEM (standard error of the mean). Data were statistically analyzed by ANOVA followed by Dunnett’s post-hoc test. Differences were considered significant when *p*-values < 0.01 (**) or < 0.001 (***). Analyses were performed using the GraphPad Prism 6 software (San Diego, CA, USA).

## 3. Results and Discussion

Two mixtures (Mix and Mix-seq) of three major phenolic compounds from olive oil (Ole, Tyr, and *p*-Cou) were prepared in order to investigate whether specific interesting molecules could arise from their simultaneous or sequential oxidation via an electrochemical technique. Firstly, the square wave voltammetry and the spectroelectrochemistry of the individual molecules were examined to access the individual data. Secondly, the phenolic standards and the mixtures Mix and Mix-seq were analyzed, before and after electrolysis, by LC-UV-ESI-MS^2^ in negative mode, in order to compare their chemical profiling. The appearance of new peaks in electrolyzed samples compared to those in the not electrolyzed samples (i.e., reference) led to identifying some specific oxidized products.

### 3.1. Square Wave Voltammetry (SWV) of the Three ArOH-EVOO Standards

**Figure 2:**
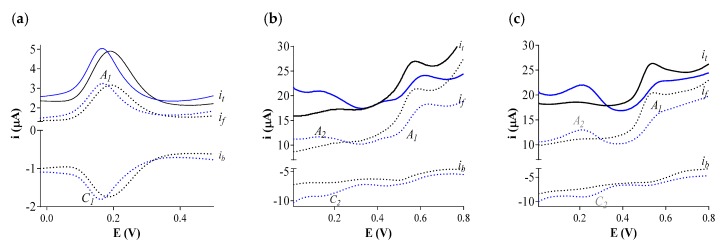
SW voltammograms of 25 µM ArOH in pH 7.4 0.1 M phosphate buffer *vs.* Ag/AgCl reference electrode using a GCE working electrode, scan rate 0.05 V s^−1^. (**a**) Ole; (**b**) Tyr; (**c**) *p*-Cou. A1: first oxidation peak; C1: cathodic peak on the reverse scan when visible. Black color: first scan; Blue color: second scan. i_t_: total current; i_f_: forward current; i_b_: backward current.

The electron transfer properties of Ole, Tyr, and *p*-Cou, were investigated by SWV in 0.1 M phosphate buffer at pH 7.4. As expected, the SWV of Ole exhibited a quasi-reversible system whereas the SWVs of the two monophenols Tyr and *p*-Cou displayed an irreversible anodic peak (*E_pa_*) at a higher potential which attests a lower electron-donating ability than Ole, respectively *E_pa_* = +0.15, +0.53, +0.56 V (Figure 2). As it is well-known, the *ortho*-diphenol function was oxidized in a two-electron-proton mechanism in its ortho-quinonic form on the anodic scan and was reversibly reduced back to the catechol form on the reverse cathodic scan [30,31] (for detailed mechanisms for phenol oxidation see [32]). The Ole SWV second scan, without cleaning the electrode surface, did not display any additional peak attesting that the quinone was stable at the voltammetry time scale. The oxidation of monophenols is monoelectronic and gives rise to a transient phenoxyl radical [30,33], (for a review on detailed mechanisms see [34]). The Tyr and *p*-Cou SWV second scan displayed an oxidation peak *A_2_* at a lower potential (*A_2_*_Tyr_ = +0.13 V; *A_2 p_*_-Cou_ = +0.20 V), related to a reduction peak, *C_2_*, at approximately the same potential, which reveals a reversible system (Figure 2b,c). As explained by Enache and al, this *A_2_*-*C_2_* system is linked to the formation of a quinone compound by water addition on a phenoxyl radical [33].

### 3.2. Spectroelectrochemical Analysis of Three ArOH-EVOO Standards Individually and in the Mix Samples

**Figure 3:**
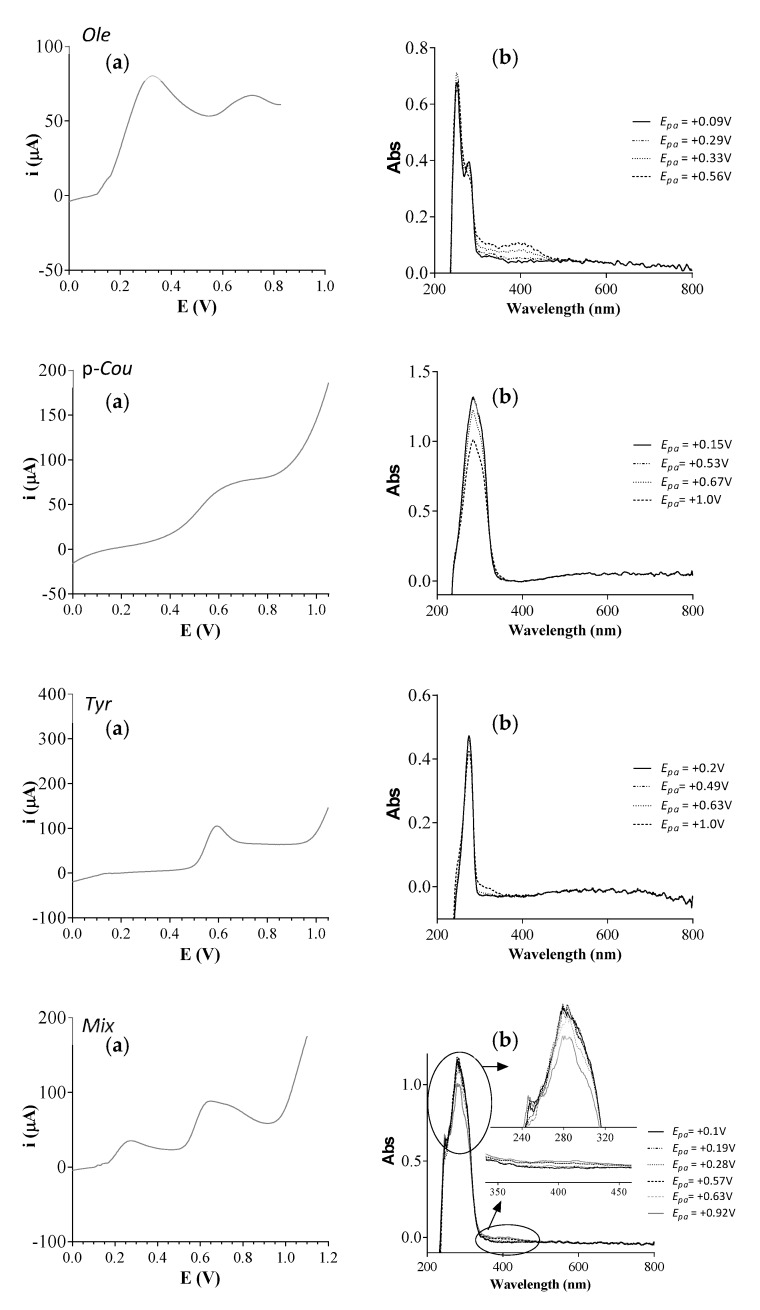
Spectroelectrochemical measurements of 2 mM ArOH and 2 mM Mix in pH 7.4 0.1 M phosphate buffer. (**a**) Linear sweep voltammetry (LSV) were analyzed at a platinum grid working electrode *vs.* Ag/AgCl reference electrode, scan rate 0.005 V s^−1^. (**b**) In situ thin layer UV-Vis spectra in the function of the potential measured during the LSV.

Controlled potential electrolysis of the ArOH-EVOO and of the mixtures (Mix and Mix-Seq) were achieved in pH 7.4 0.1 M phosphate buffer in a modified UV cell at a platinum grid electrode. UV Spectra were first recorded during the linear sweep voltammetry (LSV) at different potential values. At +0.09V (Figure 3a), Ole remained under its native form, and the wavelengths of maximum absorption λ_max_ were measured at 250 and 280 nm (Figure 3b). At +0.33 and +0.56 V, Ole was under its quinone form and an absorption band was visible at 400 nm, attesting the oxidation of the molecule. The in situ UV-Vis spectra during LSV of Tyr and *p*-Cou did not show any significant differences between before and after oxidation; nevertheless, a more pronounced hypochromic effect was noticed for *p*-Cou at 300 nm (Figure 3a,b). No transient compounds were detected here. The electrolysis of Ole, *p*-Cou, Tyr, and Mix were conducted during 7200 s at +0.7 V, +1 V, +1 V, +0.9 V, respectively (data not shown). For Ole, the UV spectrum of the final solution showed λ_max_ at 250 and 272 nm. The quinone absorption band at 400 nm was not as visible as in LSV plots, but a slight hyperchromic effect was visible at 330 nm.

For *p*-Cou and Tyr, the UV spectrum of the final solution did not exhibit a significant modification, attesting that similar chromophores were present with λ_max_ = 300 nm for *p*-Cou and λ_max_ = 275 nm for Tyr. Spectroelectrochemical measurements of 2 mM Mix showed a weak hypochromic effect at 290 nm and the appearance of a weak 400 nm band absorption on UV-Vis spectra. After electrolysis, all the solutions were retrieved, saturated with argon in a sealed vial and stored at −20 °C before LC-MS^2^ analysis.

### 3.3. The Remaining Content of the Molecules after Electrolysis Individually and in the Mix by LC-UV-ESI-MS^2^

**Figure 4:**
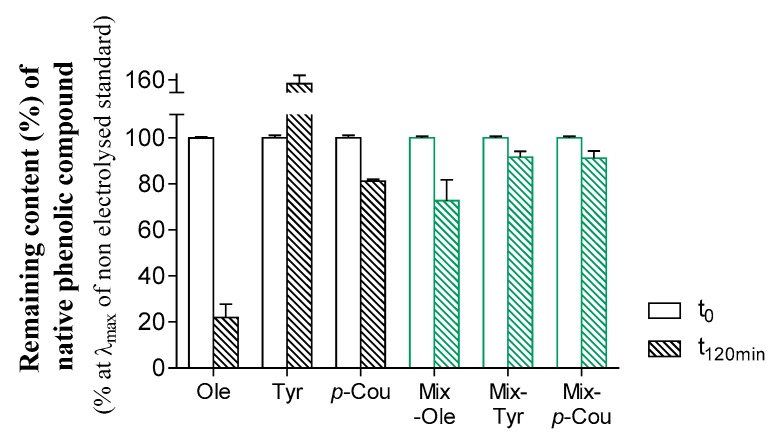
The remaining content of native ArOH after oxidation with a platinum electrode during 7200 sec at a fixed potential (E*_pa_* +0.7 V for Ole, E*_pa_* +0.90 V for the Mix, and E*_pa_* +1.0 V for Tyr and *p*-Cou). Native and oxidized solutions were analyzed by LC-UV-ESI- MS^2^. Peak areas were measured at 250 and 280 nm for Ole (tr = 38.8 min), 280 for Tyr (tr = 14.5min), and 310 for *p*-Cou (tr = 29.3 min). Green color represents the remaining content of each ArOH electrolyzed in the Mix.

In all samples except Mix-seq, the electrolysis rate of the molecules was measured from the UV-Vis area peaks in the LC-UV-ESI-MS^2^ chromatograms (Figure 4). For each standard, UV-Vis peaks were integrated at the maximal wavelength and considered 100% (i.e., reference). In the electrolyzed solutions, the peaks were integrated and expressed as a percentage of the remaining area. Surprisingly, the remaining content of Ole was higher when its oxidation was conducted in Mix rather than individually, respectively, 72% and 21%. To a lesser extent, we also observed lower oxidation in the Mix for *p*-Cou, respectively, 91% and 80%. The calculation was not possible for Tyr because the integration gave a percentage above 100% when oxidized singly putatively due to a similar absorption of some Tyr oxidized products. This Ole protection from oxidation is more likely a regeneration of the starting molecule via redox-coupled reactions. We can hypothesize that the quinone of Ole reacts in the Mix with a neoformed molecule that belongs to a reversible redox system with a lower redox potential than Ole quinone/Ole. Indeed, it is known that in a complex environment, a quinone A can oxidize an *ortho*-diphenol B belonging to another redox system with a lower potential E_redox_, which generates the resulting quinone B and regenerates the ortho-diphenol A [35,36].

### 3.4. Characterization of Products by LC-MS^2^ after the Electrochemical Oxidation of Ole

The comparison of LC-ESI-MS chromatograms of 2 mM Ole solutions, before and after electrolysis, showed the appearance of eight new compounds **O_16_**, **O_18a,b_**–**_22_**, and **O_24_** at RT 39.8, 40.3, 40.6, 41.0, 41.2, 41.6, and 42.6 min in oxidized samples (Table 1).

**Scheme 1:**
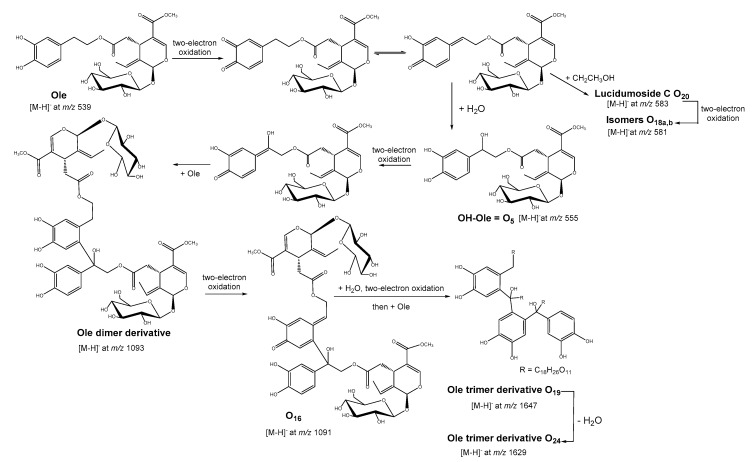
Proposed mechanism of oleuropein dimerization and trimerization after oxidation and water or ethanol addition (adapted to [2]).

Some compounds **O_16_**, **O_19_** (*m*/*z* at 1647), and **O_24_** (*m*/*z* at 1629) were generated from the Ole dimerization and trimerization after water addition and oxidation coupling steps as previously depicted by Roche et al. [2] (Scheme 1). Other trimer derivatives appeared such as **O_21_**, **O_22_**, and **O_25_** and corresponded to a loss of 28 Da (putatively a CO unit) in comparison to **O_19_** or **O_24_**. Compound **O_20_** has been already reported from olive-leaf extracts as lucidumoside C [37]. Nevertheless, in our case, this compound **O_20_** at *m*/*z* 583 probably derived from Michael’s addition on the reactive Ole quinone of ethanol used for solubilization before LC-MS analysis (Scheme 1).

**O_18a,b_** isomers at *m*/*z* 581 corresponded to the oxidation products of **O_20_** with product ions at *m*/*z* 403 and 535 characteristic of loss of glucoside or of ethanol, respectively. A significant increase of **O_1_**, **O_2_**, **O_10_**, **O_15_**, **O_17_**, and **O_25_** was also observed after Ole electrolysis compared to Ole reference in which traces of them were detected. Four of these compounds have been identified compared to literature data as 3,4-dihydroxyphenylglycol (**O_1_**) [38], 11-methyl-oleoside (**O_2_**) [11], Ole dimer (**O_15_**) [16], or Ole quinone (**O_17_**) [10]. Their occurrence could be linked to the oleuropein autoxidation process, notably **O_1_** [14] and **O_15_** [16], but their amount was significantly increased by the electrochemical oxidation as already described using other oxidation methods. Compound **O_1_** has already been reported as an oxidation product of OH-Tyr after enzymatic or Fenton reactions [14]. It was also observed after thermal or waste process of olive oil [38] like for the metabolite **O_2_** [11]. Ole quinone **O_17_** only appeared after Oleuropein Fenton or periodate oxidations [10]. It is interesting to note that two compounds **O_5_** (10-hydroxy oleuropein) and **O_14_** (oleuropein diglucoside), already present in the Ole solution reference, were observed in olive oil wastes [11] using LC coupled to electrospray-ionization mass spectrometry but here could be an artifact linked to LC-MS analysis. The Ole also demonstrated high sensitivity in water or ethanol under UV light yielding a decomposition into OH-Tyr and elenolic acid or derivatives, followed by oxidation or formation of solvent adducts [39]. **O_6_** at *m*/*z* 585 was putatively one of the examples of another Ole-ethanol adducts formed in solution. Some differences have been observed in the LC-UV-MS^2^ profiles of oxidized Ole and of oxidized Mix samples. Three peaks corresponding to three unknown compounds; **O_4_** at *m*/*z* 573, **O_8_** at *m*/*z* 617, **O_9_** at *m*/*z* 431 were detected as higher in the Mix oxidized or Mix–seq except for **O_4_** which was only in traces in the latter sample. The most remarkable results concern the presence only in traces of **O_17_**, **O_19_**, and **O_18b_** and the absence of **O_20_** in the oxidized Mix (Mix-Ox) highlighting the indirect protection against oxidation of Ole when native Tyr and *p*-Cou are present (see 2.3 for remark on redox coupled reactions leading to Ole regeneration in mixes). Indeed, these observations have been confirmed in oxidized Mix-seq, in which Ole was added to (Tyr and *p*-Cou) previously oxidized, and where these compounds, markers of Ole oxidation, were all detected (Table 1). Moreover, **O_4_** and the dimer **O_15_** were not detected in the Mix-seq after oxidation, indicating that other oxidative mechanisms occurred when the three phenols were mixed before electrolysis.

### 3.5. Characterization of Products by LC-MS^2^ after Electrochemical Oxidation of Tyr

Seven neoformed compounds labeled **T_1_**–**T_2_** and **T_4_**–**T_8_** were detected in the LC chromatograms of Tyr samples submitted to electrochemical oxidation (Table 2). It has been noted that Tyr (**T_3_**) has three *m*/*z*. None of them corresponds to the deprotonated ion, which means a potential oligomerization in the Electrospray Ionization (ESI) source. The three unknown compounds **T_1_**–**T_2_** and **T_4_** corresponded to high molecular weight compounds with *m*/*z* at 1794, 1820, and 1499 suggesting an oxidative polymerization. One dimer (**T_5_** at *m*/*z* 273), two Tyr trimers (isomers **T_6_** and **T_8_** at *m*/*z* 409), and one tetramer (**T_7_** at *m*/*z* 545) were also detected. The formation of oligomers has been already reported after enzymatic oxidation of Tyr using peroxidase in the presence of H_2_O_2_ [40]. One of these oligomers was described as a dimer with a structure arising from an ortho-ortho oxidative phenolic coupling. Chakroun and collaborators have also described the formation of Tyr dimers after enzymatic oxidation using laccase at pH 5 [41] with a structure different from those presented in [40]. The product ions of **T_5_** at *m*/*z* 255 and at *m*/*z* 243, which correspond to a loss of water and H_2_C = O unit, did not lead to select the structure of **T_5_** among the two already proposed structures. All these Tyr oligomers disappeared in the oxidized Mix, while some of them were detected in the Mix-seq samples (**T_5_** and **T_6_**). These observations could be explained by the indirect protection of Tyr oxidation in Mix. Interestingly an oligomer of Tyr has shown higher antioxidant activities compared to Tyr using DPPH assay due to the stability of its phenoxyl radical in *ortho* position [40].

### 3.6. Characterization of Products by LC-MS^2^ after Electrochemical Oxidation of p-Cou

It has been noted the presence in the reference samples of iso-*p* coumaric acid (**C_3_**), which has already been described likely due to photoisomerization [42]. Two other unknown compounds were also found in non-electrolyzed samples. The electrochemical oxidation of 2 mM *p*-Cou samples resulted in the formation of two dimers (isomers **C_4_** and **C_5_**), identified in LC-MS at *m*/*z* 325, and two decarboxylated dimers (isomers **C_7_** and **C_8_**) with *m*/*z* 281 (Table 3). The dimers have already been reported using either enzymatic oxidation [43], Fenton oxidation [10], or for all the dimers using 2,2’-Azobis(2-amidinopropane) dihydrochloride (AAPH)-induced oxidation [2]. The dimers **C_4_** and **C_5_** were not present in oxidized Mix and only in traces in Mix-seq, which suggest similar indirect protection of *p*-Cou in these samples.

### 3.7. Comparison of the Chemical Profiles between Mix and the Sequentially Electrolyzed Mix (Mix-seq)

**Figure 5:**
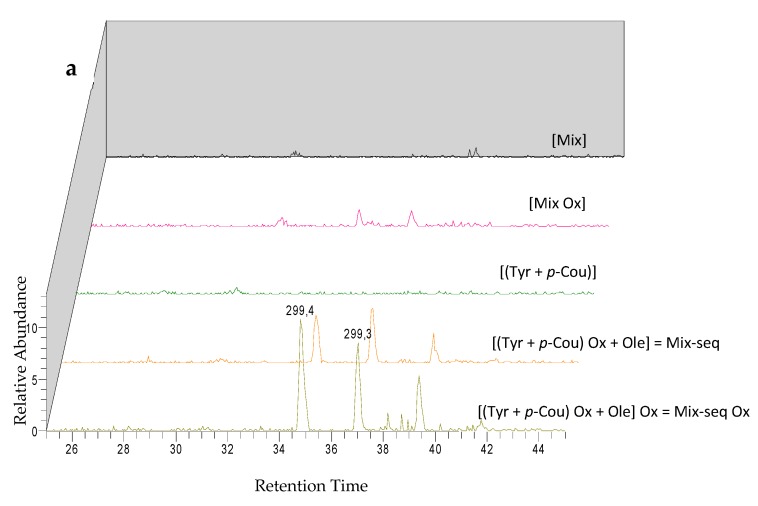

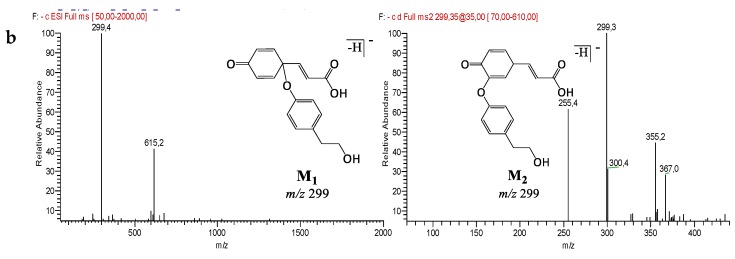
LC-ESI/MS and MS^2^ analysis in negative mode of compounds **M_1_** and **M_2_** before and after electrolysis of Mix or Mix seq. (**a**) Extracted ion chromatograms of compounds **M_1_** and **M_2_** at *m*/*z* 299. (**b**) ESI-MS spectrum of compounds **M_1_** and **M_2_** at 34.9 and 37.4 min at 35 eV collision energy.

**Figure 6:**
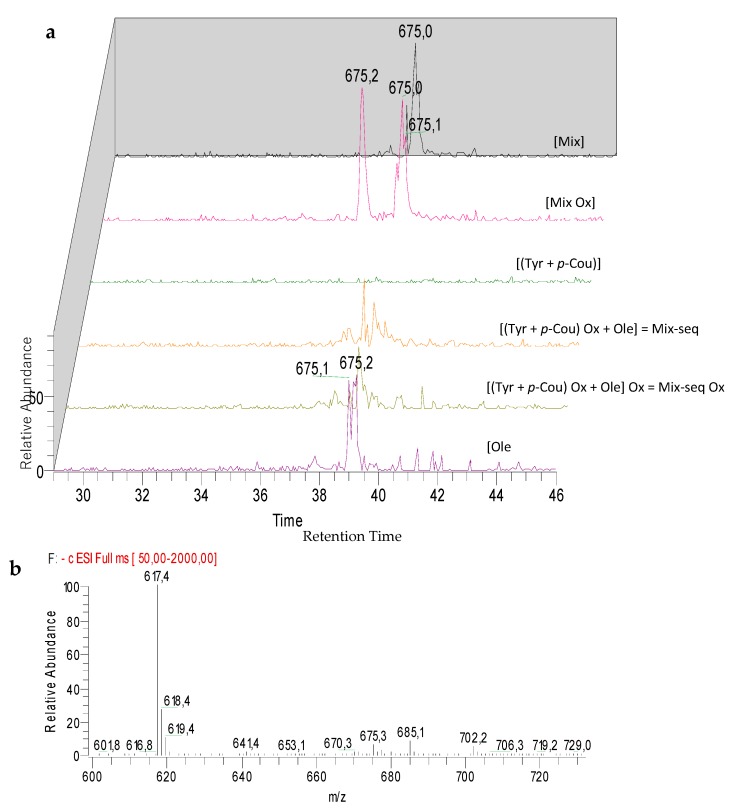
LC-ESI/MS and MS^2^ analysis in negative mode of compound **M_3_** before and after electrolysis of Mix or Ole. (**a**) Extracted ion chromatograms of compound **M_3_** at *m*/*z* 675. (**b**) ESI-MS spectrum of compound **M_3_** at 37.9 min at 35 eV collision energy.

**Figure 7:**
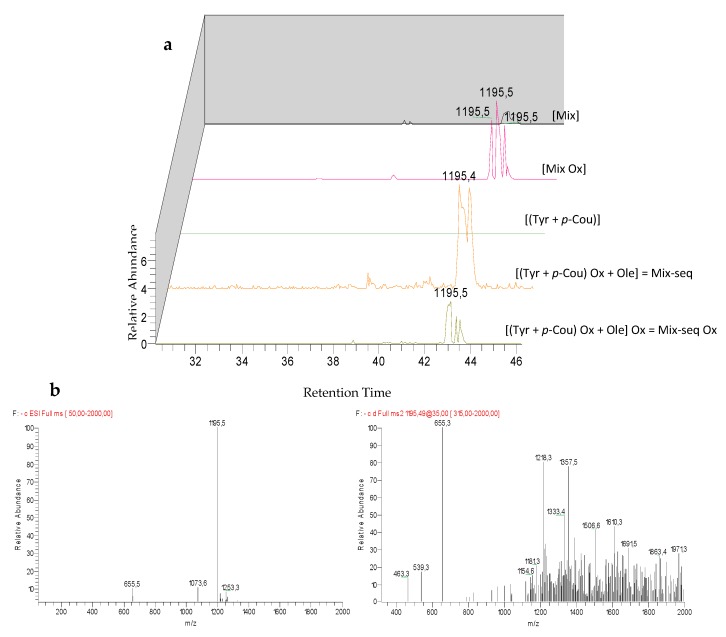
LC-ESI/MS and MS^2^ analysis in negative mode of compounds **M_4_**–**M_6_** before and after electrolysis of Mix or Mix-seq. (**a**) Extracted ion chromatograms of compounds **M_4_**–**M_6_** at *m*/*z* 1195. (**b**) ESI-MS spectrum of compounds **M_4_**–**M_6_** at 48.3, 48.4 and 48.8 min at 35 eV collision energy.

Analysis of LC-ESI-MS^2^ chromatograms of Mix samples highlighted the detection of specific peaks at *m*/*z* 299 for **M_1_** and **M_2_** (Figure 5), which could correspond to two isomers, at *m*/*z* 675 for **M_3_** (Figure 6), *m*/*z* 1195 for **M_4_**, **M_5_**, and **M_6_** (Figure 7) and at *m*/*z* 325 for unknown **M_7_** (Table 4). Except for **M_4_**, **M_5_**, and **M_6_** which were also detected in the Mix reference samples, all these compounds were observed after oxidation of Mix or Mix-seq. With respect to their product ions, we suggest that isomers **M_1_** and **M_2_** resulted from the condensation between Tyr and *p*-Cou after oxidation (Figure 5). The presence of these compounds in Mix-seq, where only Tyr and *p*-Cou were oxidized, argues in favor of this condensation. Interestingly, in the isomer **M_2_**, the unit derived from oxidized *p*-Cou is close to the structure of graviquinone, first isolated from the “silky oak” *Grevillea robusta* A. Cunn and recently described as an antitumor and oxidative-stress related metabolite of *p*-Cou methyl ester [27]. **M_3_**, which was only detected in Mix-Ox (Figure 6 and Figure S1), could be assigned to a product derived from addition between Ole and Tyr after oxidation even if the structure could not be confirmed due to the absence of MS^2^ fragmentation. This product should result from the reaction between Ole quinone, derived from Ole oxidation, and a Tyr derivative. Compound **M_4_** probably derived from Ole as some of its product ions could result from the loss of Ole moiety. In the same manner, we were unable to determine its structure. **M_5_** and **M_6_**, even if no fragmentation pathway has been observed, could be isomers of **M_4_**.

### 3.8. Neuroprotective Effect of Oxidized Ole and Mix

**Figure 8:**
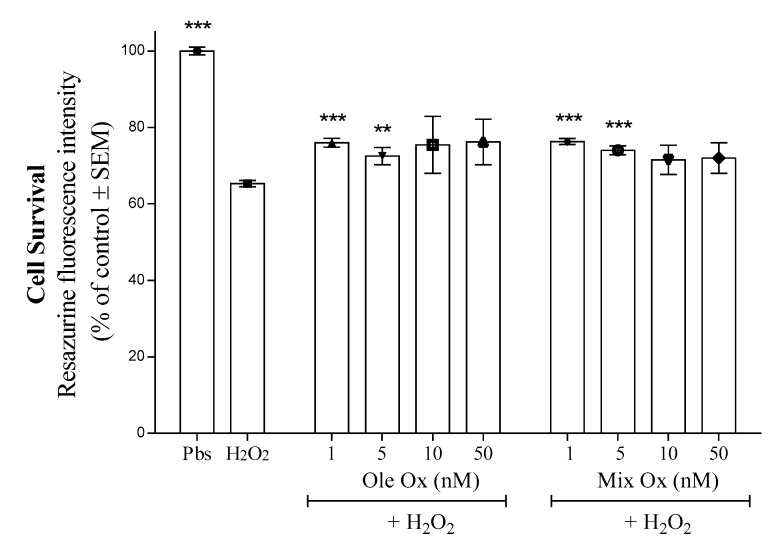
The neuroprotective capacity of the oxidized Mix (Mix-Ox) and the oxidized Ole (Ole-Ox). SK-N-SH neuronal cells were treated 24 h with H_2_O_2_ (200 µM) either in the absence or in the presence of Mix-Ox or Ole-Ox at different concentrations. Data are mean ± SEM from three separate experiments performed in sextuplicate for each group with ** *p* < 0.01 and *** *p* < 0.001, indicating a statistically significant difference versus the H_2_O_2_-treated group using Dunnett’s post-hoc test.

Our work is in line with the study of the EVOO-ArOH neuroprotective capacities and how the Mix could synergistically prevent oxidative stress-induced neuronal death, as observed in previous work [9]. With this aim, we questioned whether the oxidized compounds from the Mix (Mix-Ox) or from Ole (Ole-Ox) could prevent H_2_O_2_-induced neuronal death. Figure 8 shows that Mix-Ox and Ole-Ox were significantly able to counteract a part of H_2_O_2_ toxicity after 24h of treatment at very low concentrations (1 and 5 nM), but not at higher concentrations (10 and 50 nM). It has been noted that at these lower concentrations (1 and 5 nM), Mix did not counteract the H_2_O_2_ toxicity (Figure S2). Firstly, these results suggest that low concentrations of oxidized compounds from Mix-Ox and Ole-Ox were able to counteract, at least in part, oxidative stress-inducing neuronal death. Secondly, this efficiency depended on the concentration used.

We should highlight that the oxidized ArOH-EVOO concentration used to observe a slight neuroprotective activity was one hundred lower than the one observed in our previous work with the native Mix form (tested at 1 and 0.1 µM). Interestingly, there was no difference between the protective effect efficiency of Mix-Ox and Ole-Ox. This observation tends to show that oxidation products in Mix-Ox were as efficient as Ole-Ox, even if fewer oxidation markers of all ArOH-EVOO were present. Therefore, the neuroprotective effect could possibly due to a combination of the native ArOH and the Ole-oxidized compounds. This is in line with several studies which observed that polymerized compounds, which issue from ArOH oxidation maintained antioxidant activities [10,44], sometimes higher than their native ones [40,45,46]. Facing the low concentration range used here, we can hypothesize that the Mix-oxidation generated interesting oxidative compounds that promote the antioxidant effect through an enhancement of the electron transfer and hydrogen atom transfer capacities compared to the native compounds, as already described [44,45].

## 4. Conclusions

A spectroelectrochemical analysis of three major phenolic compounds from olive oil, either individually or in combination, was carried out in order to highlight some specific reactivities. Careful analysis of LC-ESI-MS^2^ chromatograms led to the highlighting of specific oxidized products derived from dimerization of Tyr with Ole or *p-Cou* in equimolar mixtures (Mix). The presence of these dimers could be linked with the neuroprotective effect against H_2_O_2_ toxicity observed for oxidized Mix (Mix-Ox) or oxidized Ole (Ole-Ox) at lower concentrations than for the native equimolar mixture (Mix). These results suggest the relevant role of a combination of ArOH-EVOO and their oxidized products (particularly oxidized Ole derivatives) to avoid or delay neuronal death. Taking together, these results suggest that there is a great interest in studying the influence of the ArOH-EVOO oxidation course on their neuroprotective properties and how their oxidized products could impact the neuronal redox state regulation.

## Figures and Tables

**Table 1:** Oleuropein data after LC-ESI-MS^2^ analysis of Ole, Mix, and Mix-seq before and after electrochemical oxidation.

Compound Name		RT (min)	λmax (nm)	*m/z*	Ole 2 mM		Mix 2 mM		Mix-seq 2 mM		MS^2^			
					Ref	Ox	Ref	Ox	(Tyr + *p*-Cou) Ox + Ole	Ox				
O_1_	3,4-Dihydroxyphenylglycol	4.1	280	169	traces	+	−	+	−	+	151 (−18)	123 (−46)		
O_2_	11-Methyl-oleoside	22.0	<240	403	traces	+	traces	+	+	+	223 (−180)	179 (−224)	359 (−CO_2_)	161 (−242)
O_3_	unknown	30.8		587	+	+	+	+	+	+	543 (−44)	403 (−184)		
O_4_	unknown	32.4	288	573	+	traces	traces	+	traces	-	529 (−44)	403 (−170)	222 (−350)	
O_5_	10- Hydroxy-oleuropein	35.1		555	+	+	+	+	+	+	537 (−H_2_O)	393 (162)	403 (−152)	
O_6_	Oleuropein + [EtOH]	35.8	244/307	585	+	+	+	+	+	+	523 (−62)	541 (−44)	361 (−224)	199 (−386)
											403 (−182)			
O_7_	unknown	37.0	255	615	+	+	+	+	+	+	423 (−191)	455 (−160)	273 (−344)	551 (−64)
O_8_	unknown	37.8	266/336	617	traces	traces	-	+	+	+	423 (−194)	455 (−162)	273 (−344)	585 (−32)
O_9_	unknown	38.0	266/337	431	+	traces	+	+	+	+	269 (−162)			
O_10_	unknown	38.3	267/344	567	traces	+	traces	+	-	+	373 (−194)	403 (−164)	223 (−344)	
O_11_	unknown	38.3		461	+	+	+	+	+	+	299 (−162)	446 (−15)	307 (−154)	
O_12_	Oleuropein	38.4	278	539	+	+	+	+	+	+	377 (−162)	307 (−232)	275 (−264)	
O_13_	unknown	38.6	-	553	+	+	traces	+	+	+	403 (−150)	223 (−330)	179 (−374)	
O_14_	Oleuropein diglucoside	38.6	309	701	+	+	+	+	+	+	377 (−324)	307 (394)	275 (−426)	
O_15_	Ole dimer	39.5		1077	traces	+	traces	+	traces	-	673 (−404)	813 (−264)	539 (−538)	
O_16_	Ole quinone dimer derivative	39.8	263/420	1091	−	+	−	+	traces	+	687 (−404)	403 (−688)	525 (−56)	
O_17_	Oleuropein quinone	39.9	295	537	traces	+	traces	traces	traces	+	−			
O_18a_	Oxidized lucidumoside C	40.3	259/434	581	−	+	−	+	+	+	535 (-46)	403 (−178)		
O_19_	Oxidized Ole trimer	40.3		1647	−	+	−	traces	−	+	685 (−962)	1109 (−538)		
O_20_	Lucidumoside C	40.6	255/350	583	−	+	−	−	+	+	537 (−46)			
O_18b_	Oxidized lucidumoside C	41.0	<240/412	581	−	+	+	traces	+	+				
O_21_	Ole trimer derivative	41.2	280	1619	−	+	−	+	+	+	1575 (−CO_2_)	1557 (−CO_2_, −H_2_O)	1019	
O_22_	Iso Ole trimer derivative	41.6	280	1619	−	+	−	+	+	+	1575	1557	1019	
O_23_	Iso Ole dimer	41.7		1077	+	-	+	-	traces	−	813 (−264)	673 (−404)	539 (−538)	
O_24_	Ole trimer derivative	42.6	281	1629	−	+	−	+	+	+	1019	1045	1585	1091
O_25_	Ole trimer derivative	43.6	285	1601	traces	+	−	+	traces	+	1197	793		

**Table 2:** Tyrosol data after LC-ESI-MS^2^ analysis of Ole, Mix, and Mix-seq before and after electrochemical oxidation.

Compound Name		RT (min)	λmax (nm)	*m/z*	Tyr 2mM		Mix 2mM		Mix-seq 2mM		MS^2^		
					Ref	Ox	Ref	Ox	(Tyr + *p*-Cou) Ox + Ole	Ox			
T_1_	unknown	10.4	−	1794	−	+	−	−	−	−			
T_2_	unknown	12.9	280	1820	−	+	−	−	−	−	1761 (−59)		
T_3_	Tyrosol	14.6	275	502	+	+	+	+	+	+	365 (−Tyr)	228 (−Tyr dim)	
				402	+	+	+	+	+	+	265 (−Tyr)		
				273	+	+	+	+	+	+			
T_4_	unknown	17.8	285	1499	−	+	−	−	−	−	1449 (−50)	1414 (−85)	1240 (−259)
T_5_	Tyrosol dimer	34.0	280	273	−	+	−	traces	+	+	243 (−CH_2_O)	255 (−H_2_O)	
T_6_	Tyrosol trimer	38.1	nd	409	−	+	−	traces	+	+	391 (−H_2_O)	341 (−68)	273 (−Tyr ox)
T_7_	Tyrosol tetramer	39.4	275–280	545	−	+	−	−	traces	−	409 (−Tyr ox)		
T_8_	Tyrosol trimer	40.0		409		+	−	−	−	−	271 (−neutral Tyr)	341 (−68)	136 (−Tyr dim)

**Table 3:** *p*-Coumaric acid data after LC-ESI-MS^2^ analysis of Ole, Mix, and Mix-seq before and after electrochemical oxidation.

	Compound Name	RT (min)	λmax (nm)	*m/z*	*p*-Cou 2mM		Mix 2mM		Mix-seq 2mM		MS^2^	
					Ref	Ox	Ref	Ox	(Tyr + *p*-Cou) Ox + Ole	Ox		
C_1_	*p*-coumaric acid	28.9	308	163	+	+	+	+	+	+	119 (−CO_2_)	
C_2_	iso-*p*-coumaric acid (*m*- or *o*-)	31.8	295	163	+	+	+	traces	+	+	126 (−37)	119 (−CO_2_)
C_3_	unknown	35.1	−	497	+	+	−	−	−	−		
C_4_	*p*-coumaric acid dimer	38.8	305	325	−	+	−	−	traces	traces	281 (−CO_2_)	237 (−2CO_2_)
C_5_	*p*-coumaric acid dimer	40.0	305	325	−	+	−	−	traces	traces	281 (−CO_2_)	
C_6_	unknown	48.9	−	271	+	+	−	−	+	−	213 (−58)	
C_7_	decarboxylated *p*-coumaric acid dimer	41.8	300/316	281	−	+	−	+	+	+	237 (−CO_2_)	
C_8_	decarboxylated *p*-coumaric acid dimer	42.3	292/330	281	−	+	+	+	+	+		

**Table 4:** LC-ESI-MS^2^ analysis of Mix and Mix-seq before and after electrochemical oxidation.

	Compound Name	RT (min)	λmax (nm)	*m/z*	Mix 2mM		Mix-seq 2mM		MS^2^			
					Ref	Ox	(Tyr + *p*-Cou) Ox + Ole	Ox				
M_1_	Oxidized (Tyr - *p*-Cou) dimer	34.9		299	−	+	+	+	255 (−CO_2_)	135 (−164)		
M_2_	Oxidized (Tyr - *p*-Cou) dimer	37.4		299	−	+	+	+	255 (−CO_2_)	135 (−164)		
M_3_	Oxidized (Tyr - Ole) dimer	37.9		675	−	+	−	−				
M_4_	unknown	43.3	245/284	1195	+	+	+	+	655 (−Ole)	791 (−11-methyl--oleoside)	1033 (−glu)	963
									1163 (−O_2_)	657 (−Ole Ox)	403	539
M_5_	unknown	43.4	245/284	1195	+	+	+	+				
M_6_	unknown	43.8	245/284	1195	+	+	+	+				
M_7_	unknown	48.3	305	325	−	+	+	+	281 (−CO_2_)			

## Supplementary Materials

The following are available online at https://www.mdpi.com/2218-273X/9/12/802/s1, **Figure S1:** Total ion current of Mix oxidized. **Figure S2:** Neuroprotective capacity of the Mix under its native form at the same concentrations than the observed neuroprotection with the Mix-Ox and Ole-Ox.
