# Supplementary material for: UV-Vis Spectroelectrochemistry of Oleuropein, Tyrosol, and p-Coumaric Acid Individually and in an Equimolar Combination. Differences in LC-ESI-MS2 Profiles of Oxidation Products and Their Neuroprotective Properties
Source: Biomolecules. 2019 Nov 28;9(12):802. doi: 10.3390/biom9120802 (PMC6995624; doi:10.3390/biom9120802)

biomolecules

MDPI

**Supplementary Materials:** The following are available online at www.mdpi.com/xxx/s1,

**Figure S1:** Total ion current of Mix oxidized.

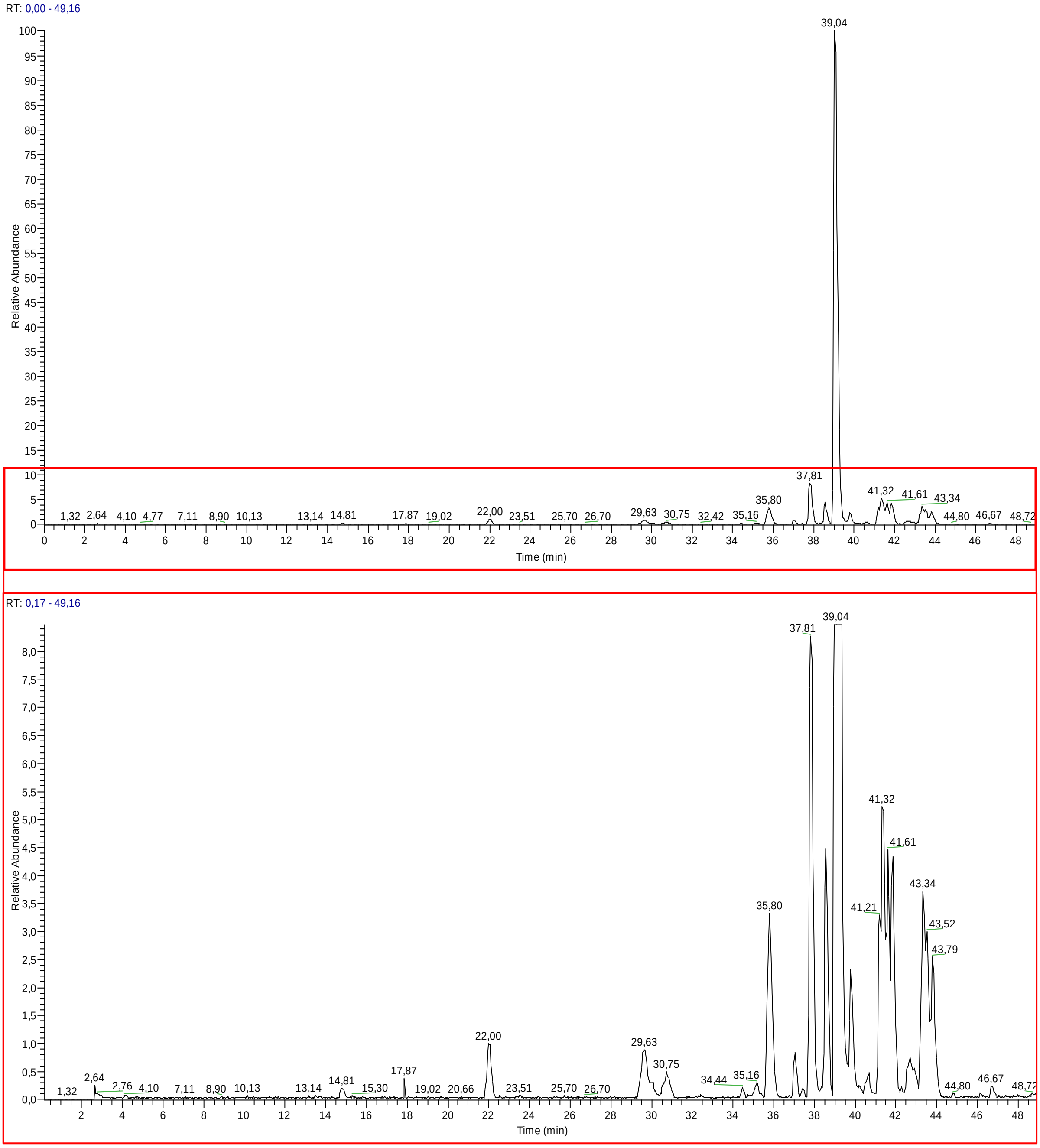

**Figure S2:** Neuroprotective capacity of the Mix under its native form at the same concentrations than the observed neuroprotection with the Mix Ox and Ole Ox.

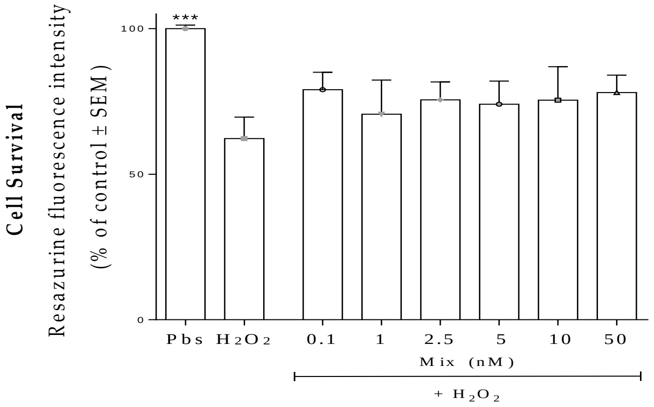

Supplement: Supplementary file 1 [file biomolecules-09-00802-s001.pdf]
